# GeneSetPheno: a web application for the integration, summary, and visualization of gene and variant–phenotype associations across gene sets

**DOI:** 10.1093/bioadv/vbaf078

**Published:** 2025-04-17

**Authors:** Jiru Han, Zachary F Gerring, Longfei Wang, Melanie Bahlo

**Affiliations:** Genetics and Gene Regulation Division, The Walter and Eliza Hall Institute of Medical Research, Parkville, VIC 3052, Australia; Department of Medical Biology, The University of Melbourne, Parkville, VIC 3010, Australia; Genetics and Gene Regulation Division, The Walter and Eliza Hall Institute of Medical Research, Parkville, VIC 3052, Australia; Department of Medical Biology, The University of Melbourne, Parkville, VIC 3010, Australia; Genetics and Gene Regulation Division, The Walter and Eliza Hall Institute of Medical Research, Parkville, VIC 3052, Australia; Department of Medical Biology, The University of Melbourne, Parkville, VIC 3010, Australia; Genetics and Gene Regulation Division, The Walter and Eliza Hall Institute of Medical Research, Parkville, VIC 3052, Australia; Department of Medical Biology, The University of Melbourne, Parkville, VIC 3010, Australia

## Abstract

**Motivation:**

The comprehensive study of genotype-phenotype relationships requires the integration of multiple data types to “triangulate” signals and derive meaningful biological conclusions. Large-scale biobanks and public resources generate a wealth of comprehensive results, facilitating the discovery of associations between genes or genetic variants and multiple phenotypes. However, analyzing these data across resources presents several challenges, including limited flexibility in gene set analysis, the integration of multipe databases, and the need for effective data visualization to aid interpretation.

**Results:**

GeneSetPheno is a user-friendly graphical interface that integrates, summarizes, and visualizes gene and variant-phenotype associations across genomic resources. It allows users to explore interrelationships between genetic variants and phenotypes, offering insights into the genetic factors driving phenotypic variation within user-defined gene sets. GeneSetPheno also supports comparisons across gene sets to identify shared or unique genetic variants, phenotypic associations, biological pathways, and potential gene-gene interactions. GeneSetPheno is a free and highly configurable tool for exploring the complex relationships between gene sets, genetic variants, and phenotypes. Target users include molecular biologists and clinicians who wish to explore a gene or gene set of particular interest.

**Availability and implementation:**

GeneSetPheno is freely accessible at: https://shiny.wehi.edu.au/han.ji/GeneSetPheno/. The source code is available on GitHub at: https://github.com/bahlolab/GeneSetPheno.

## 1 Introduction

The introduction of large-scale biobanks with extensive deep phenotypic data, combined with de-identified medical records from electronic health records (EHRs), has enabled the study of genotype-phenotype associations in hundreds of thousands of individuals (Bush *et al.* 2016). Genome-wide association studies (GWAS) and phenome-wide association studies (PheWAS) have become powerful and widely used methods for exploring associations between genetic variations and complex phenotypic traits. The integration of GWAS and PheWAS data has further enhanced our understanding of the genetic architecture underlying complex traits and diseases, generating novel hypotheses and insights into genetic and cross-phenotype relationships (Denny *et al.* 2010, Diogo *et al.* 2018, Uffelmann *et al.* 2021).

Recently, several large public data sources, such as the AstraZeneca PheWAS Portal (https://azphewas.com) (Wang *et al.* 2021, Dhindsa *et al.* 2023), FinnGen (https://r11.finngen.fi) (Kurki *et al.* 2023), and the GWAS catalog (https://www.ebi.ac.uk/gwas) (Sollis *et al.* 2023), have uncovered numerous gene-phenotype and variant-phenotype relation-ships across a broad range of phenotypes. While these resources facilitate the discovery of associations, exploring the data presents challenges, such as limited flexibility, as these resources only accept single genes or genetic variants. This highlights the need for gene set analysis to summarize gene- and variant-phenotype associations across groups of genes, as well as effective data visualization to aid interpretation. Currently, there is no interactive visualization platform that allows users to easily explore and compare these results within or across gene sets, or across resources.

With the goal of making data and results more accessible for gene sets, we developed GeneSetPheno, a comprehensive resource for analyzing gene-variant-phenotype relationships both within and across gene sets. This web application integrates data from public databases, including the AstraZeneca PheWAS Portal, FinnGen, GWAS Catalog, gnomAD (Karczewski *et al.* 2020), ClinVar (Landrum *et al.* 2014), and Human Phenotype Ontology (HPO) (Gargano *et al.* 2024) to generate visual summaries of gene, genetic variant, phenotype, and association information. GeneSetPheno provides a broader perspective on the complex relationships between genes, genetic variants, and phenotypes, helping to prioritize genes or variants for further study, and supporting the generation of biological hypotheses. In summary, GeneSetPheno provides a user-friendly interface for comprehensively exploring these associations, making it a valuable tool for diverse research applications and hypothesis generation, and could lead to the planning of bench experiments such as experimental model CRISPR approaches.

## 2 Methods

### 2.1 Implementation

GeneSetPheno is an interactive web application developed in RCoreTeam (2024), integrating a comprehensive suite of R packages, including shiny (Chang *et al.* 2024) (v1.9.1), shinythemes (v1.2.0), shinydashboard (v0.7.2), shinyWidgets (v0.8.7), rsconnect (v1.3.1), rmarkdown (v2.28), RcolorBrewer (v1.1.3), randomcoloR (v1.1.0.1), ComplexHeatmap (Gu *et al.* 2016) (v2.20.0), Genekitr (Liu and Li 2023) (v1.2.8), PhenoExam (Cisterna *et al.* 2022) (v0.1), VennDiagram (v1.7.3), rstatix (v0.7.2), ggrepel (v0.9.6), ggh4x (v0.2.8), plotly (v4.10.4), tidyverse (v2.0.0), ggpubr (v0.6.0), ggsci (v3.2.0), data.table (v1.16.0), DT (v0.33), flextable (v0.9.6), and ggplot2 (v3.5.1). This application provides robust and user-friendly functionality for integrating, summarizing, and visualizing gene and variant-phenotype associations within and across public genomic resources.

**Figure 1.**
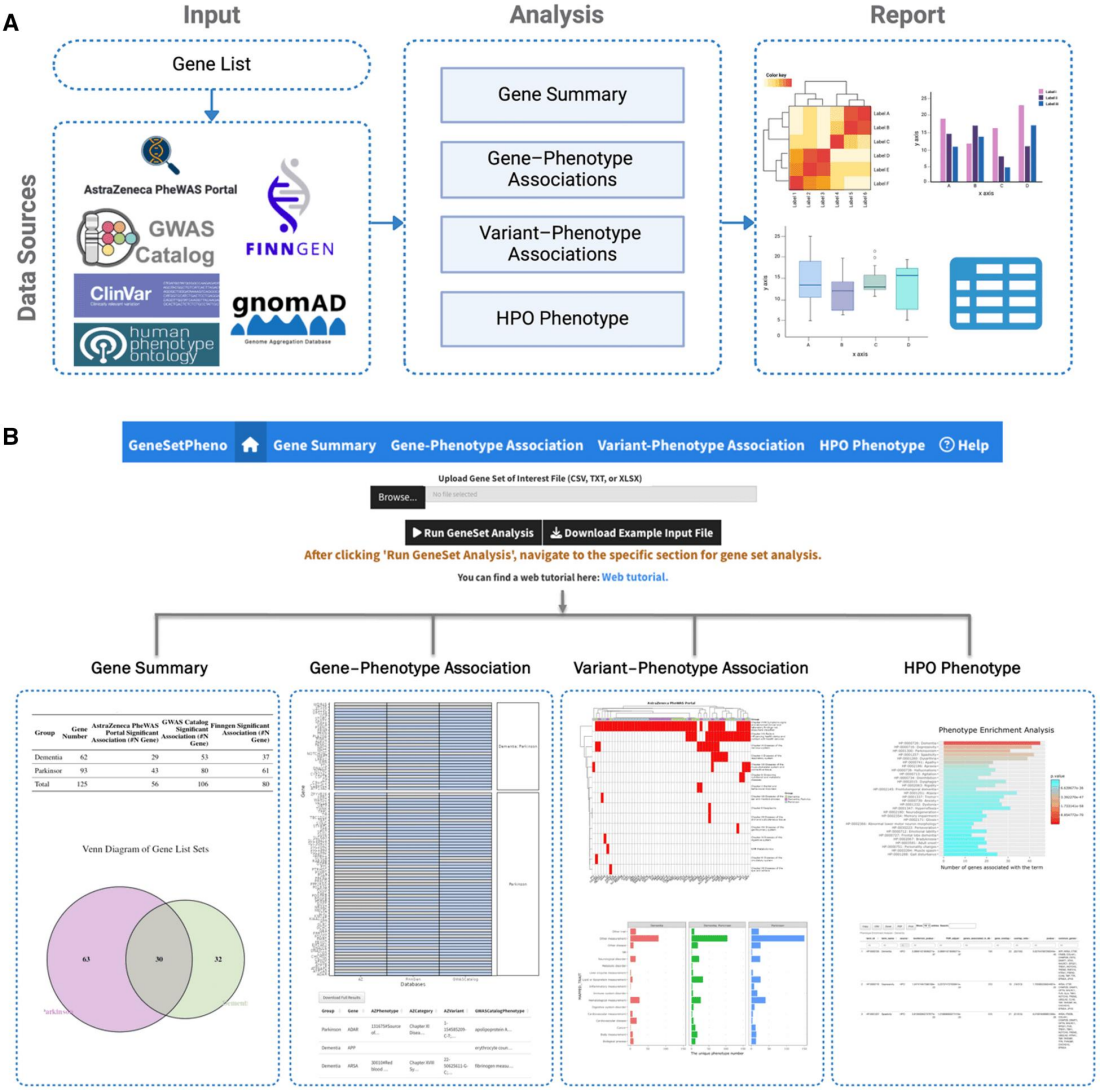
Workflow and application of GeneSetPheno. (A) The workflow of GeneSetPheno. After the input gene list is provided, GeneSetPheno runs through the Shiny framework. It efficiently loads raw data from public data resources through RShiny and then performs interactive analysis and visualization. (B) Illustration of the workflow of GeneSetPheno, highlighting its components and processes for gene set analysis.

GeneSetPheno features four modules: (1) gene summary, (2) gene–phenotype associations, (3) variant–phenotype associations, and (4) HPO phenotype (Fig. 1A). Each module integrates data and visualization components to ensure consistency and reproducibility. The application requires only a single input file (CSV, TXT, or XLSX format) containing gene list information with two columns: “Group” (representing the gene list group, such as a specific disease or condition) and “Gene” [listing gene names as approved by the Hugo Gene Nomenclature Committee (HGNC)].

### 2.2 Data retrieval and curation

GeneSetPheno integrates GWAS and PheWAS data from the AstraZeneca PheWAS Portal (AZPheWAS), FinnGen and the GWAS Catalogs. AZPheWAS evaluates associations between both common and rare protein-coding variants and phenotypes, highlighting the significant contribution of rare variants to phenotype associations. AZPheWAS summary statistics were retrieved from version 470K (v5). The complete GWAS Catalog association summary statistics (gwas_catalog_v1.0.2-associations_e112_r2024-07-08.tsv) were downloaded on 8 July 2024. To improve searchability and integration of GWAS Catalog phenotype categories, we utilized the EFO Ontology Trait Mappings (https://www.ebi.ac.uk/gwas/api/search/downloads/trait_mappings) to align the traits with their corresponding ontology terms and parent terms. FinnGen summary statistics were obtained from the most recent data freeze (data freeze 11 June 2024). Detailed information on samples, phenotypes, associations, and data processing for these databases is available in Supplementary Table S1.

Variant information, including allele frequency, clinical significance, and all the other detailed information was collected from the gnomAD v4.1 (https://storage.googleapis.com/gcp-public-data—gnomad/release/4.1/vcf/genomes/) and ClinVar (https://ftp.ncbi.nlm.nih.gov/pub/clinvar/vcf_GRCh38/) databases. These files will be manually updated with each new release of the resources, documented via the GitHub repository (https://github.com/bahlolab/GeneSetPheno).

## 3 Web interface

GeneSetPheno enables the exploration, visualization, and interaction with significant associations between gene sets and phenotypes. As a first step, users can upload their input gene list. They can then explore the different modules within GeneSetPheno (Fig. 1B).

The “Gene Summary” module generates several outputs, including a summary table, a Venn diagram showing gene counts, a table of significant gene-phenotype associations from various databases, and a visual summary of these associations (Fig. 1B). This module provides comprehensive gene set information, such as gene identifiers, genomic locations, and gene functions, with options for users to search and sort results by these parameters. Significant gene-phenotype associations from databases including AZPheWAS, the GWAS Catalog, and FinnGen are summarized, with the results presented in both tabular and figure formats.

The “Gene–Phenotype Association” and “Variant–Phenotype Association” modules offer in-depth insights into significant associations between genes, genetic variants, and phenotypes across multiple databases (Fig. 1B). These modules provide detailed information for each gene, including associated phenotypes, phenotype categories, and variants, offering a clear and concise overview of gene, variant, and phenotype associations. Additionally, a heatmap is generated to visualize the similarity between genes based on significant phenotype associations across various categories, highlighting gene clusters with common phenotype associations. These features enhance the understanding of genes, variants, and phenotypes, aiding in the identification of high-priority genes or variants for follow-up and uncovering novel relationships that offer new mechanistic insights and support hypothesis generation.

The “HPO Phenotype” module allows for the visualization of phenotype enrichment results of different gene sets using the HPO resource (Fig. 1B). It helps identify key phenotypic terms within gene sets, which is crucial for understanding the genes and effectively segregating gene sets with highly similar phenotypes.

To showcase the functionality of GeneSetPheno, we analyzed a sample gene list to explore associations between gene sets and phenotypes, focusing on two similar neurodegenerative diseases: Early-onset Dementia (EOD) and Early-onset Parkinson’s Disease (EOPD). The green gene lists, representing genes with strong clinical evidence, were obtained from PanelApp Australia (https://panelapp.agha.umccr.org; Martin *et al.* 2019). Specifically, we retrieved 62 EOD genes (Version 1.24) and 93 EOPD genes (Version 2.3), assigning them to either the “Dementia” or “Parkinson” group, with 30 genes overlapping between the two lists. A comprehensive summary of the analyses and visualizations is provided in Supplementary Section S1. Our findings highlight shared phenotype associations between these two gene lists, indicating overlapping characteristics in areas such as the nervous, behavioral, circulatory, and digestive systems, while also identifying distinct phenotypes unique to each group, including genitourinary, blood, and immune-related traits. Additionally, we summarized key gene-phenotype associations from multiple databases for each gene, enhancing our understanding of these genes. For instance, the glucocerebrosidase gene (*GBA*), a major genetic risk factor for Parkinson’s disease, also shows significant associations with blood measurements and inflammatory markers (e.g. haemoglobin concentration and C-reactive protein), the circulatory system (e.g. cardiac arrhythmias); endocrine, nutritional and metabolic diseases (e.g. disorders of sphingolipid metabolism and other lipid storage disorders), and body measurements (e.g. body mass index), indicating substantial pleiotropy.

## 4 Discussion

GeneSetPheno is an interactive web application designed for the visualization and analysis of gene sets phenotype associations. It integrates data from multiple public resources to generate comprehensive visual summaries of genes, genetic variants, phenotypes, and their associations. The web application requires only a gene set list as input, and results can be easily exported in various tabular and graphical formats. Users can explore and manage data interactively through a streamlined interface. All data resources are publicly accessible and regularly updated, ensuring that researchers have access to the latest phenotype information linked to genes. Currently, GeneSetPheno is designed for human genetic data, leveraging human-specific resources and databases. While the tool is not directly applicable to other species, similar approaches could be adapted for non-human organisms as more comprehensive cross-species genetic resources become available.

In conclusion, GeneSetPheno is the first user-friendly tool designed to analyze gene set-phenotype associations, offering enhanced functionality beyond existing tools without the need for extensive programming skills. We anticipate that GeneSetPheno will become an essential bioinformatics web server for integrating gene set-phenotype associations, supporting gene and variant prioritization, discovering novel relationships between variants, genes, phenotypes, and networks of interrelated phenotypes, offering new mechanistic insights, and fostering hypothesis generation.

## Supplementary Material

vbaf078_Supplementary_Data

## Data Availability

GeneSetPheno is freely accessible at: https://shiny.wehi.edu.au/han.ji/GeneSetPheno/. The source code and update are available on *GitHub* under the MIT license at: https://github.com/bahlolab/GeneSetPheno.
