# Supplementary material for: GeneSetPheno: a web application for the integration, summary, and visualization of gene and variant–phenotype associations across gene sets
Source: Bioinform Adv. 2025 Apr 17;5(1):vbaf078. doi: 10.1093/bioadv/vbaf078 (PMC12011357; doi:10.1093/bioadv/vbaf078)
Supplement: vbaf078_Supplementary_Data [file vbaf078_supplementary_data.pdf]

Supplementary Table

Supplementary Table S1: Detailed information for AstraZeneca PheWAS Portal, FinnGen and GWAS Catalog.

Database	Data Type	Population	Phenotypes	Significant Associations
AstraZeneca PheWAS Portal https://azphewas.com Version:470K (v5)	PheWAS	N= 419,391 European	~10K binary and ~3.5K continuous phenotypes<br><br>https://azphewas.com/phenotypeCatalogue	p ≤ 1e-8<br>Rare and common variants
FinnGen r11.finngen.fi Version:Data freeze 11	GWAS	N=453,733 Finnish	2,444 binary phenotypes (7 phenotypes were excluded in FinnGen study due to convergence issues during quality control) https://www.finngen.fi/en/researchers/clinical-endpoints	p ≤ 5e-8<br>Common variants
GWAS Catalog https://www.ebi.ac.uk/gwas/<br><br>Version:v1.0.2	GWAS<br>A curated collection of all human genome-wide association studies across multiple ancestries			p ≤ 5e-8<br>Common variants

Supplementary Section S1

Webserver submission and case studies

The GeneSetPheno webserver was designed to be biologist-oriented and to ensure compatibility with all web browsers. To demonstrate the functionality and application scope of GeneSetPheno, we applied the tool to a sample gene list dataset to explore associations between gene sets and phenotypes, focusing on two neurodegenerative diseases with similar symptoms: Early-onset Dementia (EOD) and Early-onset Parkinson's Disease (EOPD). For this example dataset, we downloaded the green gene lists, representing genes with significant clinical evidence, from PanelApp Australia (https://panelapp.agha.umccr.org). Specifically, we obtained 62 green list EOD genes (Version 1.24) and 93 green list EOPD genes (Version 2.3). The EOD panel includes genes linked to Alzheimer's disease, frontotemporal dementia, other forms of dementia, and adult-onset cognitive decline. The EOPD panel consists of genes associated with early-onset Parkinson's disease and related conditions where parkinsonism is a key feature.

Gene summary

To illustrate the different modules in GeneSetPheno, we used our example gene set list. The data for this case study can be accessed on the GeneSetPheno R Shiny help page by clicking the 'Download All GeneSetPheno Results Example' button (Figure 1A). We uploaded the example dataset for analysis, which consists of two columns: 'Group', indicating either 'Dementia' or 'Parkinson', and HGNC-approved gene names, totaling 62 EOD genes and 93 EOPD genes. Following that, we selected 'Run GeneSet Analysis' on the GeneSetPheno homepage to start the analysis.

To demonstrate the ‘Gene summary’ module in GeneSetPheno, we applied our example gene set list. Upon selecting 'Generate Gene Summary,' the following outputs will be produced: a table with detailed gene set information (Figure 1B), a Venn diagram displaying gene set counts and overlaps between groups (Figure 1C), a table summarizing significant gene-phenotype associations from multiple databases (Figure 1D), and a summary plot visualizing these associations for the gene sets (Figure 1E).

The gene set information table provides comprehensive data, including predefined group labels ('Dementia' or 'Parkinson'), gene names, genomic locations, Entrez, Ensembl, and Uniprot IDs, gene function summaries, and transcript counts. Users can query the table by gene name or other parameters, such as gene name 'APP' or group 'Dementia', and export the results in multiple formats. A Venn diagram is then displayed, summarizing the gene set group information, showing 93 genes in the 'Parkinson' group and 62 genes in the 'Dementia' group, with 30 genes overlap between the two sets. Additionally, we provide an overview of genes with significant phenotype associations across multiple databases. For example, in the AZPheWAS, 29 genes from the 'Dementia' group and 43 genes from the 'Parkinson' group show significant phenotype associations. These results can be further visualized in a heatmap, where genes and databases are represented with color coding to highlight significant associations, allowing users to quickly identify which genes are linked to specific phenotypes within each database.

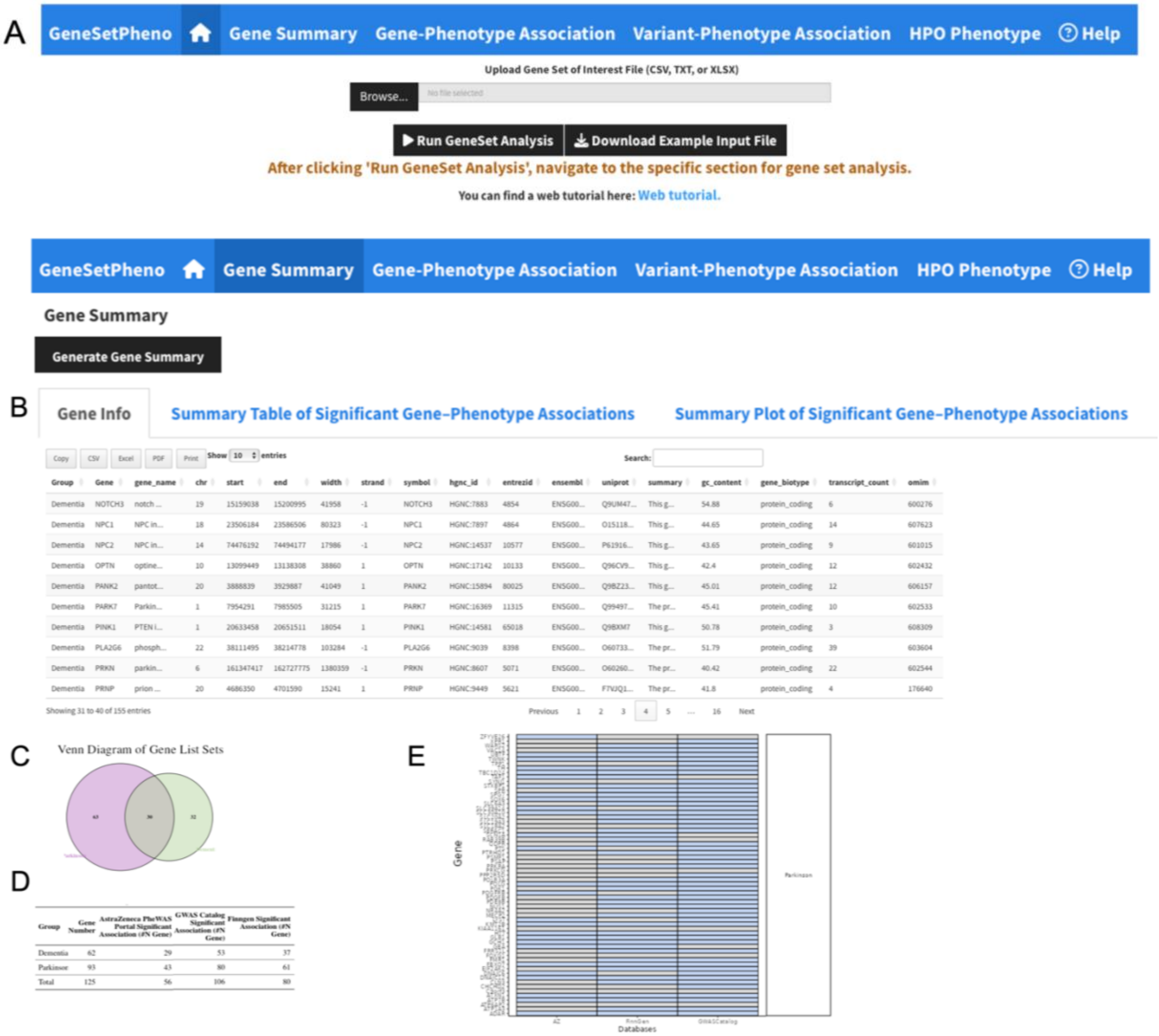

Figure 1. Overview of the GeneSetPheno 'Gene Summary' module, including its functions and results. A. Screenshot of the GeneSetPheno R Shiny homepage. B. A table presenting detailed information about gene sets. C. A Venn diagram illustrating the counts and overlaps of gene sets across different groups. D. A table summarizing significant gene-phenotype associations obtained from various databases. E. A summary plot visualizing these associations for the gene sets.

Gene–Phenotype Associations

Gene–phenotype association analysis is important for uncovering the mechanisms behind various complex human phenotypes and diseases. This module summarizes significant phenotype associations for gene sets across multiple large-scale databases, including AZPheWAS, the GWAS Catalog, and FinnGen.

This module produces two outputs upon clicking 'Gene–Phenotype Association': a summary plot and a table that displays significant gene–phenotype associations for the gene sets (Figure 2). The interactive heatmap displays significant gene–phenotype associations based on the input gene list. Users can easily access detailed information about each gene's phenotype associations across databases, such as phenotype categories, by hovering over a gene or database. For example, hovering over the triggering receptor expressed on myeloid cells-2 (*TREM2*) gene in the AZPheWAS database reveals significant associations with Chapter V: Mental and Behavioral Disorders (e.g., unspecified dementia) and Chapter VI: Diseases of the Nervous System (e.g., Alzheimer's disease-related, other degenerative diseases of the nervous system, dementia) (Figure 2A). These findings align with the inclusion of the *TREM2* gene in the 'Dementia' group list. Additionally, *TREM2* is known to play an immunoprotective role in the central nervous system in neurodegenerative diseases[1]. Beyond the AZPheWAS database, the GWAS Catalog has also identified its significant associations with neurological disorders, inflammatory measurements (e.g., C-reactive protein), and body measurements (e.g., body mass index), providing further insights into the involvement of *TREM2* in various phenotypes. In addition, the interactive summary table allows for deeper exploration by providing detailed information on gene–phenotype associations (Figure 2B). Users can access all relevant phenotype categories, phenotypes, and variants across multiple databases for each gene, offering a clear and comprehensive overview of significant gene–phenotype associations.

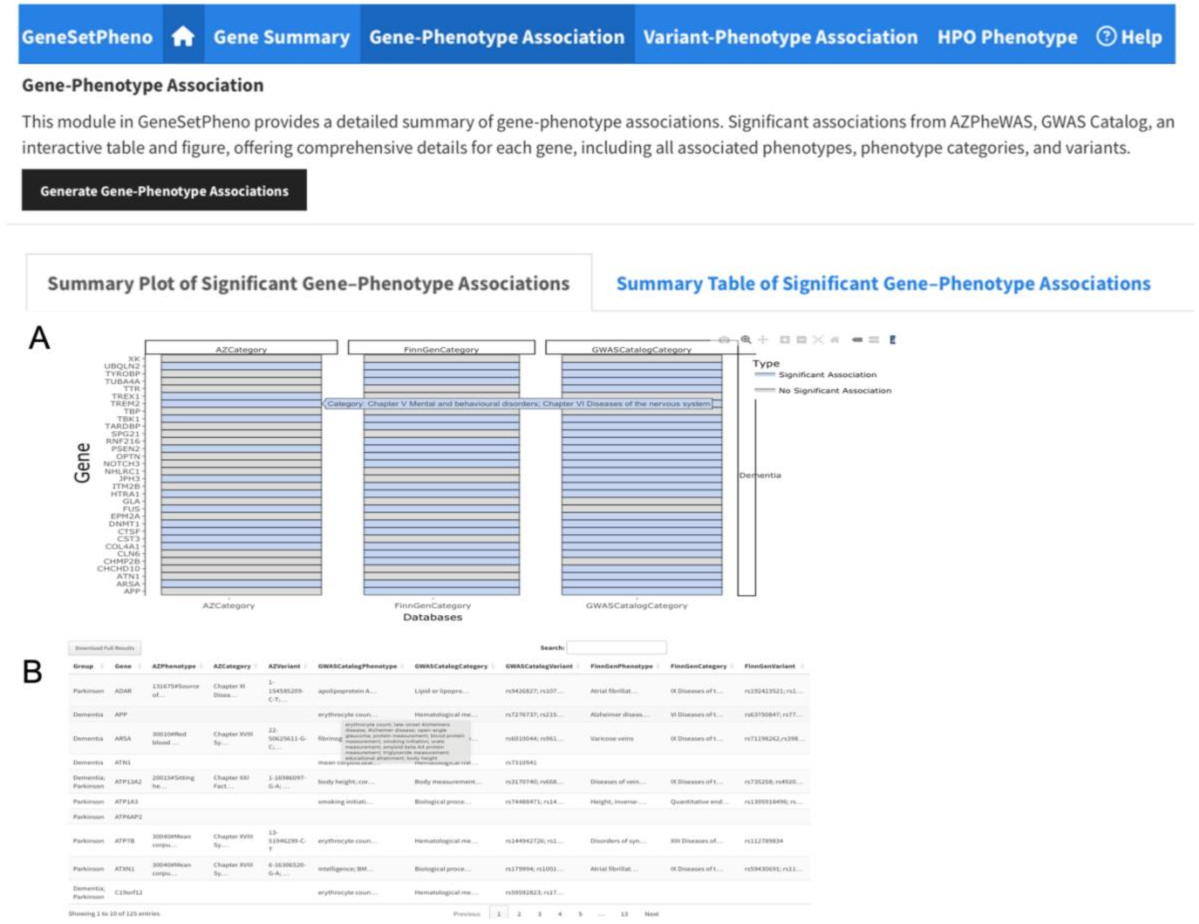

Figure 2. Overview of the GeneSetPheno 'Gene–Phenotype Association' module, including its functions and results. A. A summary plot illustrating significant gene–phenotype associations for the gene sets. B. A summary table presenting significant gene–phenotype associations for the gene sets.

Variant-Phenotype Associations

Genetic variants linked to phenotypic variability are essential for investigating how genetic changes impact biological pathways. They can reveal the regulatory mechanisms underlying complex phenotypes, leading to a deeper understanding of those phenotypes. The variant-phenotype associations module in GeneSetPheno highlights four key components: a summary table of significant variant-phenotype associations and detailed data from AZPheWAS, the GWAS Catalog, and FinnGen, reflecting their diverse phenotypic data and unique characteristics. This module serves as a comprehensive resource for showcasing significant associations between genetic variants and diverse phenotypes.

The summary table presents significant variant-phenotype associations for each variant by gene, integrating data from multiple databases (Figure 3A). It includes group labels, gene symbols, variant details (chromosome, position, reference and alternate alleles), rsID, allele frequencies from gnomAD, links to the gnomAD browser, and clinical significance from ClinVar. Additionally, the table consolidates significant phenotypes from AZPheWAS, the GWAS Catalog, and FinnGen, along with their corresponding phenotype categories. Users can search the table by variant or phenotype keyword and download the results.

Taking the AZPheWAS database as an example, this section includes three key components: phenotypic profile clustering, phenotype distribution overview, and variant-phenotype gene effect. This first generates a heatmap using the ComplexPheatmap package in R to visualize the similarity between genes based on significant phenotype associations across various categories (Figure 3B). This visualization provides an overview of genes with similar phenotypic profiles, facilitating the identification of gene clusters that share common phenotype associations.

Following this, two bar graphs are presented to show detailed phenotypic information for the 'Dementia' and 'Parkinson' gene sets. The first graph aggregates all phenotypes associated with each gene and counts the unique phenotypes within each category, separated by gene set. Hovering over a bar reveals detailed information, including associated genes, variants, and specific phenotypes within each category. The second graph, similar to the first, displays the percentage of genes in each gene set within each phenotype category, highlighting both shared distribution patterns and categories unique to each group (Figure 3C). In addition, GeneSetPheno provides a summary of the mean variant-phenotype association effect for each gene across different phenotype categories, displayed both graphically and in a table. This feature highlights the estimated gene effect within each phenotype category.

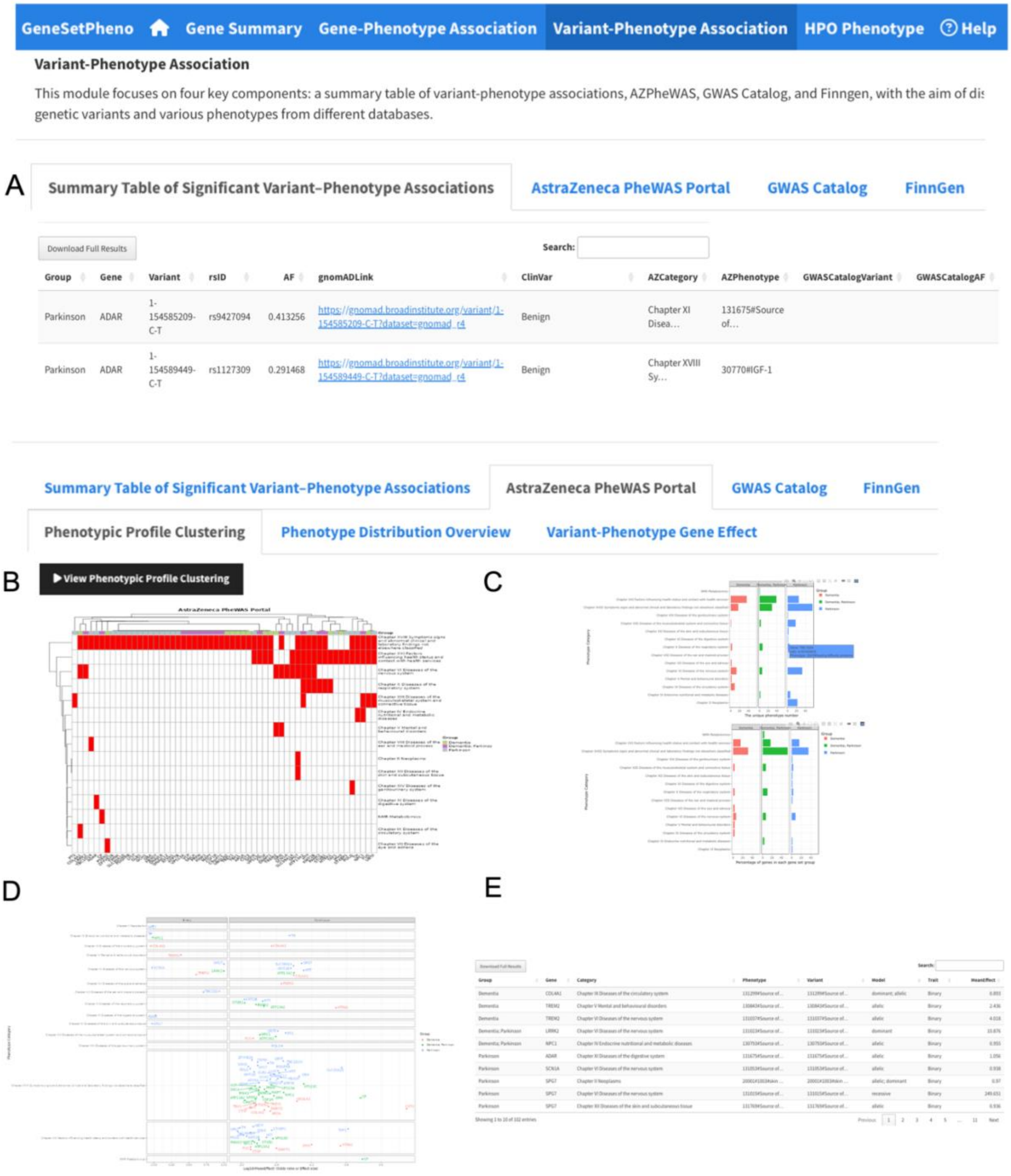

Figure 3. Overview of the GeneSetPheno 'Variant-Phenotype Association' module, including its functions and results. A. A summary table presenting significant variant-phenotype associations for each variant by gene, sourced from multiple databases. B. A heatmap illustrating the similarity between genes based on significant phenotype associations across various categories. C. A plot aggregating all phenotypes associated with each gene, counting unique phenotypes within each category and displaying results by gene set, including both gene counts and the percentage of genes in each gene set for each phenotype category. D. A summary plot depicting the mean variant-phenotype association effect for each gene across different

phenotype categories. E. A summary table detailing the mean variant-phenotype association effect for each gene across these categories.

HPO Phenotype

The HPO is a comprehensive resource that systematically defines and organizes a wide range of human phenotypic abnormalities. Identifying phenotypic terms within a gene set is crucial for gaining a deeper understanding of those genes and is also effective in segregating gene sets with highly similar phenotypes.

GeneSetPheno enables the visualization of phenotype enrichment results and comparative analysis of phenotypes across different gene sets (Figure 4). We applied GeneSetPheno to perform phenotype enrichment analysis on the 'Dementia' gene set. The results display the top enriched terms for the 'Dementia' gene set in both a plot (Figure 4A) and a summary table (Figure 4B), including terms such as Dementia (HP:0000726), Depressivity (HP:0000716), Parkinsonism (HP:0001300), Spasticity (HP:0001257), and Apraxia (HP:0002186).

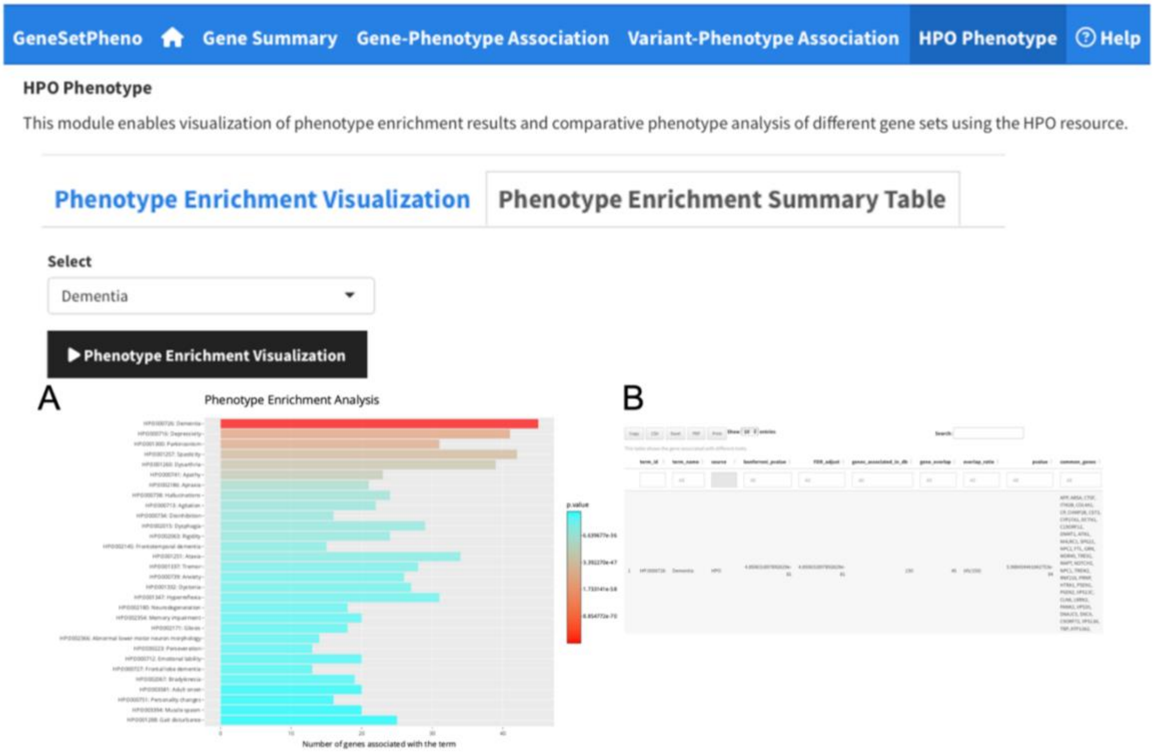

Figure 4. Overview of the GeneSetPheno ‘HPO Phenotype' module, including its functions and results. A. A plot illustrating the top enriched phenotype terms. B. A table presenting the top enriched phenotype terms.

Reference:

1. Deczkowska A, Weiner A, Amit I. (2020) The Physiology, Pathology, and Potential Therapeutic Applications of the TREM2 Signaling Pathway. *Cell*. 181(6):1207-1217.
